# The Super Anti-Browning Effect of High-Oxygen Pretreatment Combined with Cod Peptides on Fresh-Cut Potatoes During Storage

**DOI:** 10.3390/foods14091564

**Published:** 2025-04-29

**Authors:** Jiaxuan Zheng, Yishan Jiang, Aiguang Li, Mengfei Peng, Ting Wang, Runlei Kou, Ji Kang, Xia Liu

**Affiliations:** State Key Laboratory of Food Nutrition and Safety, College of Food Science and Engineering, Tianjin University of Science and Technology, Tianjin 300457, China; zhengjia_xuan@126.com (J.Z.); 18812562135@163.com (Y.J.); liaiguang202@163.com (A.L.); pengmengfei99@163.com (M.P.); wangting960718@126.com (T.W.); m13341289195@163.com (R.K.)

**Keywords:** fresh-cut potatoes slices, anti-browning during storage, short-termhigh-oxygen pretreatment, cod peptides, synergistic treatment

## Abstract

Enzymatic browning poses a formidable obstacle to the commercial sustainability of fresh-cut potatoes. Although the synergistic effects of bio-inductive technologies with natural compounds in anti-browning strategies have been observed, their full potential remains underexplored. To fulfill the demand for synergistic approaches in real-world applications, this research elucidates the complementary effects of short-term high-oxygen (HO, 80%) treatment of whole tubers in conjunction with cod peptides (CP, 0.1%) applied to fresh-cut potato slices in mitigating browning. The results demonstrated that the combined treatment (HO + CP) showed superior anti-browning efficacy compared to single treatments (HO or CP) and the untreated group (control). Specifically, peroxidase (POD) and polyphenol oxidase (PPO) activities were suppressed by 55.7% and 35.1%, respectively, under the synergistic treatment compared with the control after 8 days of storage. Meanwhile, increases in the activities of catalase (CAT), superoxide dismutase (SOD), and phenylalanine ammonia-lyase (PAL), along with an approximately 117% increase in total phenolic content, were noted with synergistic treatment. Furthermore, the combined treatment reduced malondialdehyde (MDA) accumulation by 17.5% on day 8. This effect may be attributed to enhanced antioxidant capacity and the preservation of membrane integrity. In summary, this novel strategy provides a practical synergistic solution for the control of enzymatic browning in fresh-cut potatoes.

## 1. Introduction

Fresh-cut produce is widely favored for its freshness and convenience, with annual sales exceeding $10 billion [1,2,3]. However, surface browning consistently reduces shelf life and diminishes consumer appeal of fresh-cut produce. As the fourth largest staple food in the world, over 300 million tons of potatoes are consumed per year globally, but fresh-cut potatoes are typically vulnerable to enzymatic browning [4,5].

It is well known that enzyme-mediated browning is primarily attributed to the coordinated action of polyphenol oxidase (PPO) and peroxidase (POD) in fresh-cut produce. PPO catalyzes the oxidation of the phenolic compounds to o-quinones, while POD further oxidizes these intermediates using H₂O₂ as a co-substrate, ultimately forming insoluble brown pigments. This process is triggered by cellular damage during processing, which allows enzyme–substrate contact in the presence of oxygen [6]. Meanwhile, various bio-inductive technologies and natural agents—including short-term high-oxygen exposure, UV-C light, plant extracts, essential oils, and peptides—have been identified as safe and side-effect-free alternatives to chemical anti-browning agents [7,8,9,10,11]. Notably, as abiotic stresses, typical bio-inductive technologies like heat shock, high-pressure CO_2_ treatment, and high-oxygen pretreatment are promising for their ability to activate defense mechanisms and regulate the physiological systems of fresh fruits and vegetables, but limited practical applications have been found in fresh-cut products [12,13,14,15,16]. Among these, short-term high-oxygen exposure can activate antioxidant stress responses in fruits, modulating reactive oxygen species (ROS) and the reduction–oxidation (redox) system [13,17]. Moreover, the enhanced antioxidant capacity induced by high oxygen may be linked to improved membrane integrity. The accumulation of an appropriate concentration of ROS, which act as key signaling molecules, plays a crucial role in abiotic and biotic stress sensing and activating stress-response networks [18].

Meanwhile, various proteins, peptides, and amino acids have been shown to enhance ROS scavenging activity, stabilize cell membranes, and reduce phenolic compound oxidation, ultimately delaying the browning of fruits and vegetables [11,19]. Furthermore, they directly regulate the enzymatic activities associated with the browning process. As PPO inhibitors, they impede the enzymatic conversion of phenols to o-quinones, which subsequently polymerize non-enzymatically into colored pigments [11,20]. Concurrently, POD mediates the single-electron oxidation of phenols in the presence of H_2_O_2_, a process that is also inhibited [11,21]. Additionally, phenylalanine ammonia-lyase (PAL), a crucial enzyme for phenolic biosynthesis, also experiences peptide-driven modulation [11,22]. Particularly, cod random peptides (CP) exhibit superior multi-target efficacy by simultaneously (i) inhibiting PPO activities through copper chelation and competitive binding, (ii) regulating PAL-mediated phenolic metabolism, and (iii) preserving membrane integrity via oxidative stress mitigation—as demonstrated in our preliminary studies [11]. Interestingly, bioactive peptides have beneficial effects on human health and can regulate plant dysfunctions caused by stress through the targeted modulation of cellular protein functions and protein–protein interactions [23].

ROS function as pivotal signaling entities that facilitate the rapid cellular response to various stimuli in plants. ROS are integral to the perception of both abiotic and biotic stress, the integration of diverse environmental signals, and the activation of stress-responsive pathways [24]. Mechanical processing (peeling and cutting) of fresh-cut potatoes induces abiotic stress, causing oxidative damage and metabolic imbalance. This mechanical stress inevitably triggers ROS generation in fresh-cut produce. Browning is the responding mechanism for excessive ROS, which are transferred to phenols. Hence, short-term oxygen exposure or bioactive peptide treatment could inhibit the original browning metabolic mechanism. However, limited studies have examined the synergistic effects of combining bio-inductive technologies with natural peptides.

Here, we pioneered a novel, synergistic methodology involving high-oxygen pretreatment (HO) of whole potatoes, followed by cod peptide application on fresh-cut slices. To investigate the efficacy of the combined treatment in inhibiting browning, we evaluated its impact on color and visual characteristics, key enzyme activities related to enzymatic browning, antioxidant capacity, browning substrate content, malondialdehyde (MDA) content, and membrane permeability in fresh-cut potatoes stored at 4 °C.

## 2. Materials and Methods

### 2.1. Raw Materials and Sample Preparation

*Solanum tuberosum* potatoes (Netherlands 7) were purchased from a local agricultural market in Tianjin, China. The selected tubers exhibited uniform maturity, consistent size (5–7 cm diameter), and intact surfaces (free of mechanical damage or greening), with postharvest storage duration limited to <1 week. A total of 120 tubers were used in the experiment, comprising 3 biological replicates × 4 treatments × 10 tubers per treatment. The acceptable samples were stored at 4 °C until further processing. The cod peptides were procured from Qingdao Ruikang Biotechnology Co. Ltd., Qingdao, China.

The selected potatoes were divided into four groups. Two groups were placed in a hermetically sealed gas chamber with a cuboid shape that was equipped with a gas cylinder inlet at one end and a gas composition analyzer (PBI-Dansensor A/S, Ringsted, Denmark) at the opposite end. An initial flush with nitrogen gas was introduced into the sealed chamber to displace ambient air. Subsequently, oxygen was infused into the chamber until the concentration reached 80%. The potato samples were then exposed to this atmosphere level for 20 min. Concurrently, the other two groups of potatoes were kept under ambient air conditions, characterized by an oxygen concentration of 21%, for an equivalent duration of 20 min.

Following the gas treatments, all potatoes were cleansed with tap water, manually peeled, and sectioned into slices (0.5 cm). Two groups (separately exposed to 80% oxygen and ambient air) were immersed in distilled water, while the other two groups were soaked in a CP solution (0.1% *w*/*w*). After a 5-min immersion period, the potato slices were gently dried using sterile gauze.

The treated potato slices were then placed in polyethylene-sealed pouches, sized 280 mm × 180 mm, which were labeled control, 0.1% CP, 80% O_2_, and 0.1% CP + 80% O_2_ according to their treatments. Then, they were stored at 4 ± 1 °C for 8 days. Characteristics of the fresh-cut potato samples were assessed at two-day intervals.

### 2.2. The Color of Fresh-Cut Potatoes

Colorimetric attributes were quantified using a calibrated colorimeter (Model WG-0218, Beijing Fanghua Technology Institute, Beijing, China) as browning quantitative indicators [25]. Before measurement, the instrument was calibrated with black and white standard tiles. Color values were recorded as lightness (L*), red-green (a*), and yellow-blue (b*) values. The color difference between the initial samples (zero time) and storage-aged samples were contrasted by calculating the total color difference (∆E*), as shown in Equation (1):

(1) $$∆E*=\sqrt{{\left(∆L*\right)}^{2}+{\left(∆a*\right)}^{2}+{\left(∆b*\right)}^{2}}$$

### 2.3. The Activity of PPO

A previously established protocol with slight adjustments for practical applications was followed [26]. Initially, 3.0 mL buffer (0.1 mol L^−1^ acetic acid-sodium acetate, pH 5.5) was added to a mortar containing 3.0 g of samples. Rapid homogenization into a slurry was conducted under ice bath conditions, followed by centrifugation of the generated slurry at 10,000× *g* for 30 min at 4 °C using a refrigerated centrifuge (Model TGL-16M, Hunan Xiangyi Laboratory Instrument Development Co., LTD, Changsha, China).

For the enzymatic assay, 4.0 mL of acetic acid-sodium acetate buffer (0.05 mol L^−1^, pH 5.5), 1.0 mL of catechol solution (0.05 mol L^−1^), and 100.0 μL of the collected supernatant were combined and incubated at refrigeration temperature. Absorbance values at 420 nm were then recorded at one-minute intervals over a six-minute period using a UV–visible spectrophotometer (Model UV-800B, Shanghai Mapada Instruments Co., Ltd., Shanghai, China). PPO activity was measured in units per gram of fresh weight (U g^−1^).

### 2.4. The Activity of POD

POD activity was quantified based on an adapted methodology [27]. The extraction procedure for this sample was identical to that used for the PPO extract. A reaction mixture was then prepared, containing 3.0 mL of guaiacol solution (0.025 mol L^−1^), 0.2 mL of H_2_O_2_ solution (0.5 mmol L^−1^), and 0.5 mL of the collected supernatant. After a 15-min incubation period, absorbance at 420 nm was measured at one-minute intervals, and POD activity was expressed as U g^−1^.

### 2.5. The Activity of CAT

A revised protocol was used to measure CAT activity [28]. Two-gram aliquots of each sample were homogenized in CAT buffer (5.0 mL) and then centrifuged (10,000× *g*, 30 min). Subsequently, 0.1 mL of the resultant supernatant was combined with hydrogen peroxide (2.9 mL, 20 mmol L^−1^), and absorbance at 240 nm was recorded at 30-s intervals for a total span of 3 min. Deionized water was the baseline control. The unit of CAT activity was expressed as µmol min^−1^ g^−1^.

### 2.6. The Activity of SOD

SOD activity was quantified based on its ability to inhibit 50% of nitroblue tetrazolium (NBT) reduction [29]. Two-gram aliquots of each sample were homogenized in 5.0 mL of sodium phosphate buffer (0.1 mmol L^−1^, pH 7.8) containing DTT (5 mmol L^−1^) and PVP (5%). The homogenate was subjected to centrifugation (12,000× *g*, 30 min). The mixture comprised 1.7 mL of sodium phosphate buffer (50 mmol L^−1^, pH 7.8); 300 µL each of metformin (130 mmol L^−1^), NBT (750 µmol L^−1^), EDTA-Na_2_ (100 µmol L^−1^), and riboflavin (20 µmol L^−1^); and 100 µL of the supernatant. The blend was incubated for 15 min under fluorescent light. Following the reaction, absorbance was measured at 560 nm, and the enzymatic activity was expressed in U g^−1^.

### 2.7. The Activity of PAL

A modified protocol was used for measurement [30]. Aliquots of 3 g from each sample were blended in 3.0 mL of boric acid buffer (0.05 mol L^−1^, pH 8.8). This mixture was then centrifuged (10,000× *g*, 30 min) at 4 °C. Then, 0.5 mL of the supernatant was introduced to a 10-min pre-incubated solution of boric acid buffer (3.0 mL, 0.05 mol L^−1^, pH 8.8) and L-phenylalanine (0.5 mL, 0.02 mol L^−1^). Absorbance readings were taken immediately and after a 60-min incubation period at 37 °C, with readings taken at 290 nm. The activity was expressed in U g^−1^.

### 2.8. Total Phenolic Content

The total phenolic content was quantified using an adapted Folin’s phenol reagent procedure [13]. Two-gram aliquots of each sample were homogenized in 60% ethanol (5.0 mL) and centrifuged (10,000× *g*, 10 min). Subsequently, 0.25 mL of the supernatant was relocated and shielded from light. This was subsequently mixed with 0.25 mL of Folin’s phenol reagent, 0.8 mL of Na_2_CO_3_ solution (20% *w*/*w*), and 5 mL of distilled water. Following a 25-min incubation under light-avoidant conditions, the 760 nm absorbance was logged. The data were normalized to fresh weight and expressed in g kg^−1^, using gallic acid equivalents as a reference.

### 2.9. Membrane Permeability

Membrane permeability was assessed by measuring the relative conductivity using a DDS-11A conductivity meter (Shanghai, China) [27]. Uniform 8 mm in diameter potato slices (2.0 g) were prepared using a hole punch from randomly selected fresh potato samples. These slices were incubated in distilled water and agitated for 10 min. After decanting the used water, the slices underwent three washes with distilled water before being placed in a thermostatic shaker for incubation (one hour with 20 mL of new distilled water). Conductivity at this stage was recorded as $p_{1}$. The solution was then boiled (100 °C, 15 min) using an electric thermostatic water bath (Model HB-458350, Shenzhen Tiannan Dibei Industrial Co., Ltd., Shenzhen, China) and re-measured for conductivity $p_{2}$ after rapid cooling to room temperature. Membrane permeability was calculated using Equation (2) as follows:

(2) $$\gamma =\left(p_{1}-p_{0}\right)/\left(p_{2}-p_{0}\right)\times 100\%$$

where $p_{0}$ is the electrical conductivity of distilled water.

### 2.10. Malondialdehyde Content

The malondialdehyde (MDA) content was quantified using a modified version of a previously established protocol [25]. Two-gram aliquots of each sample were ground into a homogenate using 10.0 mL of 100 g L^−1^ trichloroacetic acid solution and quartz sand. The homogenate was moved to a centrifuge tube for centrifugation (10,000× *g* for 20 min). Then, 1.0 g of the supernatant was combined with 1 mL of 0.6% (*w*/*w*) thiobarbituric acid solution and boiled for 20 min. Upon reaching around 20–25 °C, the blend was centrifuged (10,000× *g*, 15 min) again. Thereafter, the pooled supernatants were analyzed spectrophotometrically at 450 nm, 532 nm, and 600 nm. The MDA content was quantified and reported in units of μmol kg^−1^ fresh weight.

### 2.11. Antioxidant Capacity

The antioxidant capacity was assessed using the 1,1-diphenyl-2-picrylhydrazyl (DPPH) radical-scavenging assay [31]. Homogenized potatoes (5.0 g) were centrifuged (10,000× *g*, 15 min) at 4 °C. Subsequently, three mixtures were prepared and incubated under light-proof conditions for 30 min: (1) DPPH solution (2.0 mL, 257.7 mg L^−1^) and ethanol (0.5 mL, 95% *w*/*w*); (2) DPPH solution (2.0 mL, 257.7 mg L^−1^) and supernatant (0.5 mL); and (3) supernatant (0.5 mL) and ethanol (2.0 mL, 95% *w*/*w*). The optical absorbance at 517 nm for each mixture was recorded as $A_{0}$, $A_{s}$, and $A_{r}$, respectively. The capacity to scavenge DPPH radicals was calculated using Equation (3):

(3) $$\gamma =\left[1-\left(A_{s}-A_{r}\right)/A_{0}\right]\times 100$$

### 2.12. Statistical Analysis

The experimental design for evaluating fresh-cut potatoes incorporated five distinct treatment conditions and an untreated control group. Each condition was assessed at 0-, 2-, 4-, 6-, and 8-day time points after treatment, with each assessment replicated three times daily. One-way analysis of variance (ANOVA) was used for statistical analysis, employing SPSS 24.0 software with a significance threshold of *p* ≤ 0.05.

## 3. Results and Discussion

### 3.1. Changes in Color and Overall Visual Characteristics

**Figure 1:**
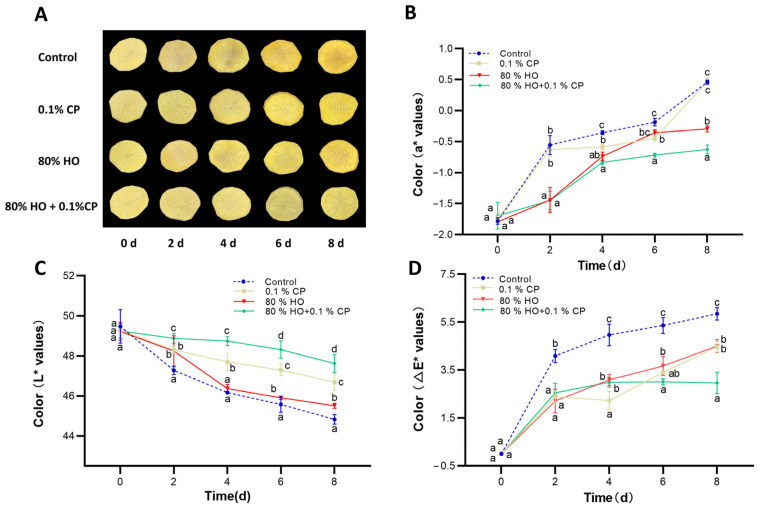
Effects of high-oxygen combined with cod peptide (HO + CP), high-oxygen alone (80% HO), or cod peptide alone (0.1% CP) treatments on the overall visual quality and color alterations of potato slices during storage at 4 °C. (**A**) Photographic representations, (**B**) a* values, (**C**) L* values, and (**D**) ΔE* values are displayed as the average of three distinct replicates. Mean values ± standard deviation (SD) (*n* = 3) are depicted. Distinct letters denote noteworthy dissimilarities among treatments for each sampling period (*p* ≤ 0.05).

The changes in color and visual characteristics of fresh-cut potatoes with different treatments stored at 4 °C for 8 days are shown in Figure 1. The effect of 80% HO pretreatment, 0.1% CP, and the combined treatment all positively affected the overall visual characteristics and a* value (red index) for surface browning (Figure 1A). Especially, the HO and 0.1% CP combined treatment showed the most prominent anti-browning effect of visual characteristics during the entire storage period and possessed the lowest a* value after 4 days of storage (Figure 1B). Consistent with the results, the combined treatment showed an obvious promotion effect on the L* value (brightness index) during the entire 8 days of storage (Figure 1C). Compare to the control, all treatments exhibited a lower overall color change, and ∆E* displayed the least color deviation after 4 days of storage (Figure 1D).

Previous studies have shown that individual treatments with 0.1% CP or 80% HO both exhibit anti-browning effects [11]. Here, the combined treatment showed the best anti-browning effect, which was consistent with the results of ultrasound combined with natural extracts as well as HO combined with supercooled storage [31,32,33]. The likely explanation could be the combined physiological impact of HO and 0.1% CP on fresh-cut potatoes. Therefore, the synergistic use of physical and natural agents presents a promising anti-browning approach for the fresh-cut industry.

### 3.2. PPO, POD, and PAL Activities and Total Phenolic Content

**Figure 2:**
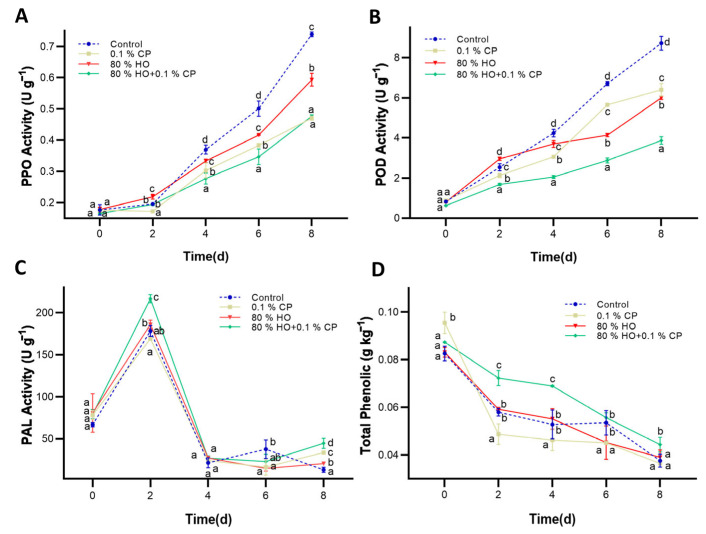
Effects of high-oxygen combined with cod peptide (HO + CP), high-oxygen alone (80% HO), or cod peptide alone (0.1% CP) treatments on the (**A**) polyphenol oxidase (PPO) activity, (**B**) peroxidase (POD) activity, (**C**) phenylalanine ammonia-lyase (PAL) activity, and (**D**) total phenol content of potato slices during storage at 4 °C for 8 days. The values are displayed as the average of three distinct replicates. Mean values ± standard deviation (SD) (*n* = 3) are depicted. Distinct letters denote noteworthy dissimilarities among treatments for each sampling period (*p* ≤ 0.05).

As is well known, enzymatic browning is influenced by cellular damage, substrate concentration, and enzyme activity [34]. The key regulatory enzymes and substrates associated with anti-browning metabolism are shown in Figure 2. The upward trends in PPO and POD activities were suppressed by HO + CP, only 80% HO, or only 0.1% CP, except for the first two days of only 80% HO treatment, in fresh-cut potatoes (Figure 2A,B). Among the three treatments, the combined treatment group showed the slowest rise of PPO activity after two days of storage, and the value was 35.14% less than that in the control group on day 8. Meanwhile, the POD activity in the combined treatment group remained at the lowest level during storage, measuring 55.65% below the control on day 8. Oddly, the HO treatment showed notably increased PPO and POD activities during the first two days due to the stress response, but this surge was tempered by the co-application of CP in the combined treatment group.

In addition, as the primary enzyme involved in phenolic compound metabolism, PAL activity was excellently enhanced after HO + CP treatment during storage, regardless of fluctuations in its activity (Figure 2C). Both as a browning substrate and an antioxidant, the total phenolic content consistently decreased during fresh-cut potato storage (Figure 2D). Notably, despite minimal differences between individual treatment groups and the control, the combined treatment group consistently maintained the highest phenolic content due to the synergistic effect of HO stress and peptides, with values 117% higher than the control samples on day 8.

Various scholars have documented the decrease in PPO and POD activities through the synergistic effect on natural anti-browning agents and concurrent physical treatments [31,32,35]. The lowest activities of both PPO and POD were observed in the combined treatment. Oddly, the combined treatment modulated POD activity more distinctly than PPO in later stages. From day 4 onward, the suppression of POD activity reached levels comparable to the additive effects of individual treatments. By day 8, POD activity in the CP, HO, and combined treatment groups was reduced by 26.6%, 31.2%, and 55.6%, respectively, relative to the control. Simultaneously, potentially because of the highest PAL activity, the highest accumulation of total phenol content occurred and was advantageous for mitigating oxidative damage [36,37]. Traditionally, anti-browning agents in fresh-cut products fall into three models during storage: one model increases total phenolic content while reducing PPO, POD, and PAL enzyme activities; the second model decreases total phenolic content while enhancing PPO and POD activities; and the third model reduces all four aforementioned factors [38,39,40]. Here, the integration of HO with CP established a novel model, suppressing PPO and POD activities, while augmenting PAL activity and total phenol content in the combined treatment group.

Evidence suggests that peptides can inhibit the activities of PPO and POD by interacting with the active sites of the enzymes, inducting competitive inhibition [11]. For PPO, anti-browning agents interact with its copper sites [41]. In addition, certain amino acids in peptides, such as L-cysteine, can interact with phenolic substance sites, resulting in non-competitive inhibition [42]. Alternatively, a subset of peptides might interact with quinones to produce colorless chelating compounds [43]. The 80% HO treatment may indirectly influence the activities and substrate accessibility of PPO and POD by regulating H_2_O_2_ production, and peptides address initial excessive oxygen stress caused by HO as a complement [23,44]. Specifically, hyperoxia treatment increased PPO and POD activities above control levels on day 2, while the synergistic effect of cod peptides mitigated this short-term overstimulation, resulting in 11.2% and 5.0% lower PPO and POD activities, respectively, in the combined treatment group compared to hyperoxia treatment alone. The results here suggest that HO pretreatment may enhance the effectiveness of subsequent peptide-based interventions.

### 3.3. Antioxidant Capacity (DPPH) and ROS Scavenging Capacity (CAT and SOD Activities)

**Figure 3:**
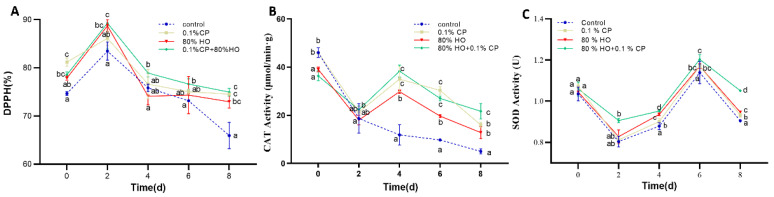
Effects of high-oxygen combined with cod peptide (HO + CP), high-oxygen alone (80% HO), or cod peptide alone (0.1% CP) treatments on the (**A**) antioxidant capacity, (**B**) catalase (CAT), and (**C**) superoxide dismutase (SOD) of potato slices during storage at 4 °C for 8 days. They values are displayed as the average of three distinct replicates. Mean values ± standard deviation (SD) (*n* = 3) are depicted. Distinct letters denote noteworthy dissimilarities among treatments for each sampling period (*p* ≤ 0.05).

The samples with combined treatment consistently showed higher DPPH free radical scavenging rates compared to the control group (Figure 3A). Remarkably, the combined treatment improved the relatively poor free radical scavenging capacity of the samples with HO pretreatment on day 4. In addition, as the principal enzymatic antioxidants involved in mitigating oxidative stress, SOD and CAT collaborate to maintain stable levels of ROS within plant cells [45]. Despite the fluctuating decline in CAT activity of the samples during storage, the combined treatment group consistently exhibited relatively high CAT levels (Figure 3B). The observed fluctuations in catalase (CAT) activity can be attributed to the inherent temporal dissociation between (1) the initial ROS-mediated signaling that triggers antioxidant demand and (2) the subsequent molecular processes involving CAT gene transcriptional activation and translational enzyme biosynthesis. Moreover, the SOD activity of fresh-cut potatoes, while fluctuating within a defined range, exhibited elevated levels in the combined treatment group (Figure 3C). The observed fluctuations in superoxide dismutase (SOD) activity likely reflect a biphasic regulatory pattern involving (1) initial enzymatic depletion during the oxidative stress phase, followed by (2) subsequent activity restoration mediated by compensatory biosynthesis. Hence, the combined treatment synergistically enhances the effects of HO stress and CP during the storage of fresh-cut potatoes.

Reactive oxygen species (ROS) are vital signaling molecules mediating plant responses to abiotic and biotic stresses [24]. Here, the short-term high-oxygen treatment served as an abiotic stress that activated cellular responses to peeling and slicing through ROS signaling. The process sets off adaptive mechanisms to counteract potential damage and triggers defense responses [46,47]. Concurrently, cod peptides enhance the antioxidant capacity of the potatoes when incorporated at optimal concentrations into the combined treatment. The peptides are rich in various amino acids with antioxidant properties, particularly tyrosine and tryptophan, which neutralize ROS and mitigate hydroxyl radical damage induced by H_2_O_2_, thereby providing cellular protection against oxidative stress [35,48]. Notably, peptides can mitigate the excessive stress response induced by HO [49]. It is speculated that these cod peptides function not only as electron donors in radical quenching but also act to stabilize reactive species by terminating free radical chain reactions. Therefore, the combined treatment effectively combines the short-term adaptive effects of high-oxygen pretreatment with the antioxidant properties of cod peptides, synergistically offering multilayered protection.

### 3.4. Membrane Permeability and Lipid Peroxidation Indicator (MDA Content)

**Figure 4:**
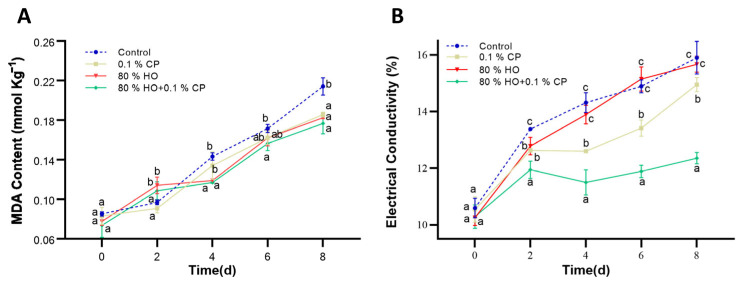
Effects of high-oxygen combined with cod peptide (HO + CP), high-oxygen alone (80% HO), or cod peptide alone (0.1% CP) treatments on the (**A**) malondialdehyde (MDA) content, and (**B**) relative electrical conductivity of potato slices during storage at 4 °C for 8 days. The values are displayed as the average of three distinct replicates. Mean values ± standard deviation (SD) (*n* = 3) are depicted. Distinct letters denote noteworthy dissimilarities among treatments for each sampling period (*p* ≤ 0.05).

Lipid peroxidation, a central contributor to cellular membrane damage, produces MDA as a byproduct, which serves as a biomarker for assessing the degree of lipid peroxidation [14]. The MDA content of fresh-cut potatoes increased over time during storage (Figure 4A). Despite a sharp rise in MDA levels on day 2 in the two groups treated with HO due to high-oxygen-induced oxidation, both exhibited a plateau phase following this rise. The MDA accumulation in the combined treatment group had significantly decreased by 17.50% compared to the control on day 8. Furthermore, the combined treatment group maintained the lowest relative electrical conductivity during storage, indicating reduced membrane permeability and, thus, less cellular damage (Figure 4B).

Although short-term exposure to high-oxygen conditions may induce some ROS production, it stimulates the plant’s adaptive defense mechanisms, inhibiting the accumulation of MDA and other oxidative stress markers [50]. In parallel, 0.1% CP significantly regulated cell integrity. Consequently, the combined treatment of 80% HO and 0.1% CP exhibits synergistic antioxidative effects in fresh-cut potatoes, reducing MDA content and alleviating cellular membrane damage, thereby inhibiting the browning process. In a word, the combined treatment showed markedly synergetic effects, which may establish the defense system and fortify the resilience of fresh-cut potato slices.

### 3.5. Correlation Analysis

**Figure 5:**
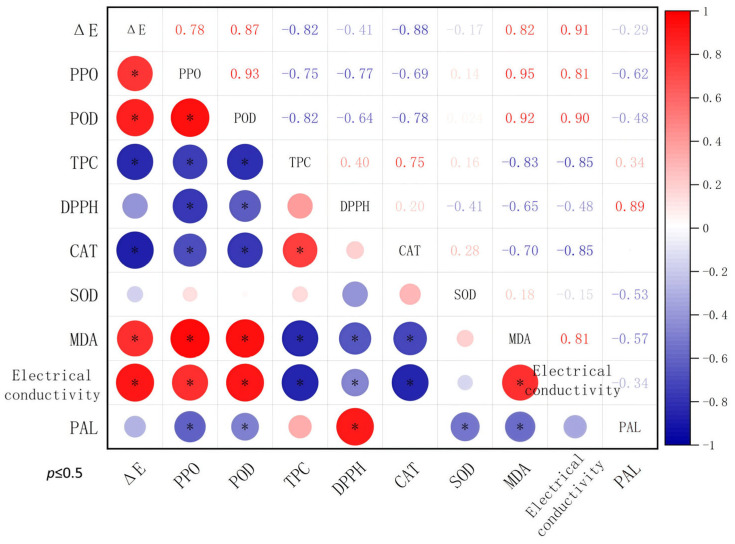
Correlation heatmap depicting relationships between ΔE* values and physiological/biochemical parameters. Color scale represents Pearson’s correlation coefficients (r), with red indicating positive correlations and blue indicating negative correlations. The asterisks in the figure denote statistically significant correlations (* *p* ≤ 0.05). The size of each circle corresponds to the absolute value of Pearson’s correlation coefficient (|r|), where larger diameters denote stronger correlations.

As is well known, PPO and POD are key enzymes involved in browning pigment production, and POD and PAL are key enzymes related to stress responses. Although both PPO and POD showed correlations with ΔE*, POD activity exhibited a stronger correlation with color change (r = 0.87 *) than PPO (r = 0.78 *) (Figure 5). The correlation between POD and ΔE* was higher than that of PAL (r = −0.28). Meanwhile, as the critical enzyme in the redox system, CAT showed strong correlations with browning (r = −0.88 *). Oddly, the activity of SOD showed a relatively modern correlation with browning. As a marker of oxidative damage, MDA accumulation showed a positive correlation with ΔE*, indicating that cellular oxidative damage is linked to browning. More importantly, membrane integrity, which is reflected by relative electrical conductivity, demonstrated the strongest correlation with ΔE* (r = 0.91 *). Furthermore, total phenolic content was negatively correlated with ΔE*, confirming phenolics as key substrates for browning reactions [34].

The correlation analysis among different indicators illustrated that cellular damage was the central driver of browning. The correlation index of PPO, POD, and PAL elucidated that POD achieves browning regulation in response to short-time high-oxygen stress and CP treatment. This may occur because high-oxygen treatment induces ROS accumulation (H_2_O_2_), and POD was effectively enhanced because it helps to alleviate oxidative stress by eliminating H_2_O_2_ [24]. For the redox system, CAT played a more pivotal role in mitigating browning, also possibly because it is essential for efficient H_2_O_2_ scavenging [51]. In summary, the electrical conductivity, POD and CAT activity, and MDA accumulation responses contribute to the anti-browning effects of HO + CP treatment in fresh-cut potato storage.

## 4. Conclusions

In conclusion, we report for the first time the effect of short-term high-oxygen pretreatment (80% HO) with cod peptide (0.1% CP) application after slicing on the anti-browning of potatoes. The results demonstrated that the combined treatment maintained the best visual appearance; the lowest PPO and POD activities; and the highest PAL, CAT and SOD activities. Furthermore, this treatment showed both the highest total phenolic content and the lowest MDA accumulation, indicating superior antioxidant capacity. Cell membrane integrity was also better preserved in the samples treated with HO + CP. Collectively, the short-term high-oxygen pretreatment combined with 0.1% cod peptide treatment provides an industrial strategy of simple, safe, low cost and convenient method that holds great promise on anti-browning of fresh-cut potato.

## Data Availability

The authors declare that they have no known competing financial interests or personal relationships that could have appeared to influence the work reported in this paper.
